# A systematic review of interventions aiming to improve newly-qualified doctors’ wellbeing in the United Kingdom

**DOI:** 10.1186/s40359-022-00868-8

**Published:** 2022-06-26

**Authors:** Aditya Krishnan, Opeyemi Odejimi, Ian Bertram, Priyamvada Sneha Chukowry, George Tadros

**Affiliations:** 1 Birmingham Heartlands Hospital, University Hospitals Birmingham NHS Foundation Trust, Birmingham, UK; 2 Psychiatric Liaison Team, Birmingham and Solihull Mental Health Foundation Trust, Birmingham, UK; 3 Royal Army Medical Corps, Birmingham, UK; 4 Aston Medical School, Aston University, Birmingham, UK

**Keywords:** Junior doctor, Wellbeing, Intervention, United Kingdom, Intervention, Systematic review, Physician, Newly-qualified doctor, Stress, Burnout

## Abstract

**Background:**

Newly-qualified doctors in the United Kingdom experience a great deal of stress and have poor wellbeing when compared to more senior counterparts. A number of interventions have been put in place to boost healthcare professionals’ wellbeing, but little is known about interventions aimed to improve the wellbeing of newly-qualified doctors in the United Kingdom. This study aims to systematically review current evidence of interventions which improved the wellbeing of newly-qualified junior doctors in the United Kingdom.

**Methods:**

Five key electronic databases were searched. Subsequently, reference scanning and citation search was performed. Studies were included if they were conducted from the commencement of the Foundation Programme in 2004, until 2019. In addition, studies had to be performed on junior doctors: working in the United Kingdom and within their first five years post-qualification and have a quantitative outcome. Studies which did not meet these criteria were excluded. Quality was assessed using the modified Newcastle-Ottawa scale. Bias was not formally assessed using a standardised tool.

**Results:**

Seven papers met the inclusion criteria and identified three main types of interventions: mentorship, mindfulness and clinical preparation interventions. The majority of included studies reported a positive result from the performed intervention, suggesting these to be beneficial in improving junior doctor wellbeing, and thereby reducing anxiety and stress levels. However, most of the studies used small sample sizes.

**Conclusions:**

This review reveals that there is dearth of evidence on the effectiveness of intervention to improve the wellbeing of newly-qualified doctors in the United Kingdom. Most of the identified interventions focused on relieving stress and anxiety inherent within newly-qualified doctors’ training programmes. However, wellbeing interventions need to take into cognisance all the factors which impact on wellbeing, particularly job-related factors. We recommend that future researchers implement large-scale holistic interventions using appropriate research methods.

*Systematic review registration*: PROSPERO CRD42019127341.

**Supplementary Information:**

The online version contains supplementary material available at 10.1186/s40359-022-00868-8.

## Background

Burnout and psychiatric morbidity has been identified to be prevalent and worryingly high amongst doctors in the United Kingdom (UK) [1]. Over the years, the percentage of doctors from the UK Foundation Programme (UKFP) applying directly for further training has significantly reduced, with just over a third applying for core training; for instance, 34.9% applied for core training in 2019 compared to 71.3% in 2011 [2, 3]. Junior doctors have been noted to have high levels of burnout and stress due to a myriad of factors including increased workload, poor training opportunities and rota gaps [4]. In fact, there have been many incidents of high-profile suicides recorded amongst junior doctors, particularly in comparison to more senior counterparts [5, 6].

In this review, we define newly-qualified doctors as those working within the first five years after qualification from medical school. Medical graduates in the UK go on to complete the two-year Foundation Programme as Foundation Year (FY) doctors in National Health Service (NHS) hospitals; most then proceed on to three- or four-year Core Training (CT) programmes in various specialities, before pursuing further specialist training as “higher trainees” prior to becoming consultants.

Whilst the term “junior doctor” refers to any doctor in training, the scope of this review is limited to newly-qualified doctors due to the difficulty of transition in their work environment, and frequency of work-related stress reported [7]. Furthermore, the challenges faced by these newly-qualified doctors are understandably different to those faced by senior trainees [8], many of whom may be up to eight years into their medical careers, despite still being labelled ‘junior doctors’ [9].

The concept of wellbeing is a broad idea, and the challenge of a suitable definition is acknowledged by some authors [10, 11]. It is often said that wellbeing encompasses an individual’s general satisfaction with their personal lives, sense of purpose and social functioning; not simply the absence of disease. In this review we define wellbeing as broader satisfaction in life, feelings of control and a sense of purpose rather than short-term gratification which may be transient. Evidence demonstrates that being actively engaged in full-time employment is beneficial to wellbeing; however, aspects of work such as lack of autonomy or poor senior support can damage an individual’s wellbeing [12].

The determinants of wellbeing are multifactorial, including aspects of the individual’s physical and mental health, sense of fulfilment from work, social inclusivity and quality of living environment [13]. Governments and employers frequently often target various determinants under their control. These may be targeted interventions focused on those identified to have poor wellbeing, or preventative interventions aimed to maintain wellbeing of the workforce [14]. Examples of these include mentorship and counselling services. Interventions to improve employee wellbeing have been poorly studied, with previous reviews identifying generally poor-quality evidence in the UK for occupational interventions which improve wellbeing of staff [15].

A 2009 study into staff wellbeing within the NHS found increased stress and poorer wellbeing within its workforce compared to other governmental organisations [16]. Junior doctor wellbeing has reportedly suffered for a myriad of reasons, but largely attributed to poor staffing making it difficult to organise leave, a busy workload and a disconnect between managers and newly-qualified doctors [17]. Several NHS organisations recognise the need for interventions and support services to promote wellbeing and reduce psychological distress and burnout for junior doctors. However, there are uncertainties about the effectiveness of these types of interventions. The aim of this systematic review is to examine evidence about interventions to improve wellbeing of these newly-qualified junior doctors in the United Kingdom.

A number of previous systematic studies have been performed on the wellbeing of healthcare professionals, with many focusing on physicians; however, none have exclusively focused on newly-qualified doctors, especially junior doctors in the UK. For instance, a recent 2020 systematic review performed in the UK on interventions designed to minimise mental illness in doctors focused on its impacts on patient care rather than the wellbeing of the physicians themselves [18].

Furthermore, other systematic reviews performed in the UK and other countries which investigated physician wellbeing interventions did not identify any studies from the UK on junior doctors [19–24]. Also, other reviews which focused on wellbeing amongst staff in particular specialties did not distinguish newly-qualified doctors in their analyses [25–27]. The lack of existing literature in this area, and the necessity of this topic in ensuring wellbeing of newly-qualified junior doctors demand this review to be performed. This is the first systematic review reporting wellbeing interventions specifically on junior doctors in the UK; in particular, focusing on junior doctors within the first five years after qualification.

### Research questions

- What wellbeing interventions are in place for newly-qualified doctors in the UK?
- How effective are the available wellbeing interventions for newly-qualified doctors in the UK?
- What are the current gaps in research about wellbeing interventions for newly-qualified doctors in the UK?

## Methods

The development of this systematic review was documented in a protocol (PROSPERO ID: CRD42019127341) [28], and developed in line with the Preferred Reporting Items for Systematic Reviews and Meta-Analyses (PRISMA) Checklist [29].

### Inclusion and exclusion criteria

This systematic review included studies performed on junior doctors working in the United Kingdom and within their first five years post-qualification (particularly FY and CT doctors). A wellbeing intervention is defined by the authors as any process or action taken with the primary intention to improve the broader satisfaction of an individual. Hence, it can range from activities such as mentorship programmes, induction, group therapy and many more aimed to promote job satisfaction, feelings of control and a sense of purpose for newly-qualified doctors. We included any primary research study design measuring a quantitative outcome, including mixed-methods studies. In cases where studies reported the use of a validated diagnostic tool, validation was based on self-reported declaration of the original authors of the included papers.

Only English-language studies conducted between August 2004 and December 2019 were included. This period was selected to include studies from the commencement of the pilot year of the Foundation Programme in 2004. Additionally, we recognise the upsurge of wellbeing interventions during the first wave of the COVID-19 pandemic. These were carried out to mitigate the unprecedented stress on the health service during the pandemic; however, we experientially note many of these were aimed to address stress and burnout specifically during the COVID-19 pandemic. We hence chose 2019 as an end-point to exclude interventions designed specifically for and during the COVID-19 pandemic, and to represent the ‘normal’ (i.e. non-COVID) pattern of work. Unpublished works and studies failing to meet the above criteria were excluded.

### Search strategy

**Fig. 1:**
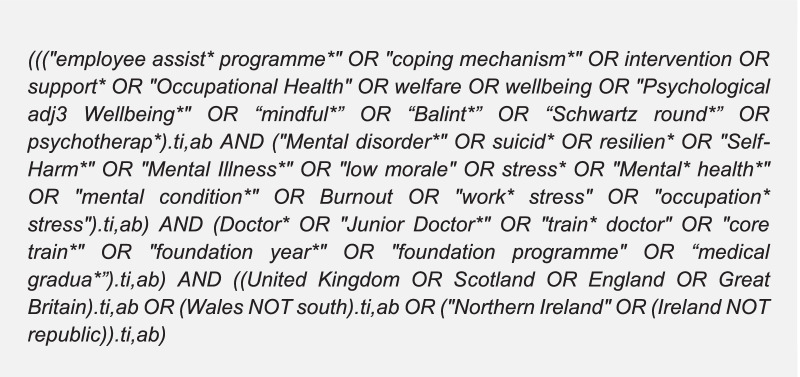
Search strategy for PubMed

Five key databases (EMBASE, PsycINFO, PubMed, CINAHL and MEDLINE) were electronically searched on 04 February 2019 for English-language studies conducted between 2004 and 2019. An updated search was run on 15 October 2020 using the same inclusion criteria and dates; this was to ensure that studies which were published in 2019, but not indexed in the databases until later were still included in the review. The search strategies used MeSH and text terms, boolean operators and truncation. Where possible, journal thesaurus keywords were included. Figure 1 shows one such search strategy. Full search strategies are included in Additional file 1: Appendix 1. We subsequently scanned reference lists and performed citation searches (Web of Science) to further identify sources not indexed in these databases. Title screening was performed manually by two independent reviewers, with any discords resolved through discussion. Abstract screening was subsequently performed by the two reviewers using the same manual method as title screening.

We accessed PROSPERO to identify existing, ongoing and previously conducted systematic reviews exploring junior doctor wellbeing. Several existing protocols on wellbeing were identified, of which two stated aims similar to ours; however, our inclusion and exclusion criteria differ from theirs. The first by Petrie et al. explores interventions improving the mental health of physicians, regardless of grade or country of origin with a focus on interventions preventing mental health disorders in physicians without pre-existing psychiatric conditions [27]. Their key outcomes of interest were anxiety, depression and suicidal ideation, whereas our protocol includes non-psychiatric wellbeing too, such as stress management. Additionally, their proposal includes studies where physicians make up a minimum of 70% of the population. Their study population therefore included an unspecified number of non-physicians.

The other protocol by Webb and Fraser aimed to identify quantitative and mixed-methods studies which examine the effectiveness of interventions that enhance or maintain junior doctor wellbeing [30]. However, we found no updates to their proposal since January 2018. Moreover, Webb and Fraser did not explicitly define their use of the term ‘junior doctor’, whereas our protocol focuses on newly-qualified junior doctors within their first five years post-qualification. Furthermore, Webb and Fraser’s inclusion is limited to studies that used a validated measurement tool for wellbeing, whereas our review does not exclude studies which use unvalidated tools.

Also, Webb and Fraser’s protocol excludes studies which involve participants with previously diagnosed mental health problems, whereas our protocol includes participants with pre-existing psychiatric conditions. Indeed, excluding doctors with pre-existing mental health conditions may exclude up to half of all participants [31]. To the best of our knowledge, there are no systematic reviews that have investigated the impact of interventions aiming to improve the wellbeing of junior doctors within the first five years post-qualification.

### Data extraction

Data from included studies were extracted, and this includes: author name, year of publication, sample size, type of study, recruitment strategy and methodology. In addition, demographic variables, intervention details and results were extracted. Missing data were documented as such, and no assumptions were made. Data extraction was conducted by two reviewers on a spreadsheet to collate and compare findings, with disparities resolved through discussion. Data extraction included: sample size, study design, recruitment strategy, gender and grade breakdown of participants, department of study, screening tool used to identify wellbeing parameters, intervention performed, presence of pre-existing conditions, duration of data collection, duration of intervention, outcomes measured, stages for data collection and results. A data extraction summary is provided in Additional file 1: Appendix 4 and Additional file 2.

Quality was assessed by two independent reviewers using the modification of the Newcastle-Ottawa Scale (mNOS) produced by Odejimi et al. [32]. Assessment for quality was independently performed by two reviewers, and discrepancies were discussed to achieve consensus. The mNOS scale assessed three domains (Appendices 5 and 6): selection, comparability, and outcomes using a ten-point scale. Odejimi et al. proposed an eight-point scale rather than the original ten-point scale used by Herzog et al. to exclude points for exposure risk which are not relevant to this systematic review [33].

## Results

### Search outcome

**Fig. 2:**
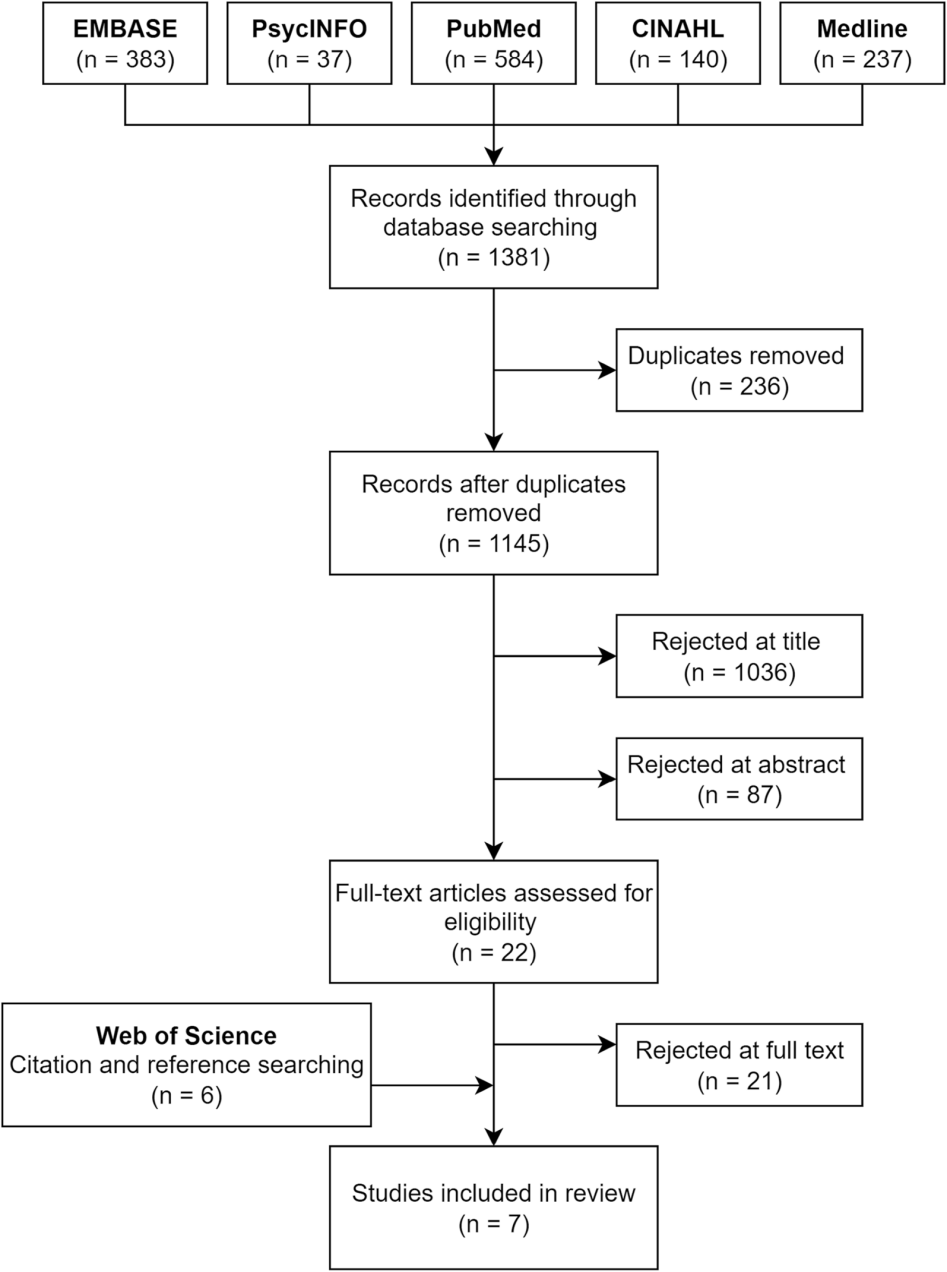
PRISMA flow demonstrating outcome from search

Systematic search produced 1381 results from the five databases, of which 236 were duplicates (Fig. 2). After removal of duplicates, the remaining 1145 studies were assessed for suitability by screening the title and abstract. Following title screening, 1036 papers were excluded because they either referred to an irrelevant population (N = 819), such as non-medics, patients or senior doctors, or were conducted outside the 2004–2019 time-frame specified in this review (N = 189). Additionally, studies which did not collect quantitative data after performing an intervention in the UK (N = 28) were excluded.

A further eighty-seven papers (N = 87) were excluded at abstract screening. The most common reason for an article to be rejected at abstract screening was the study not performing any intervention on participants (N = 63). Twenty-two full articles (N = 22) were retrieved and assessed against the inclusion criteria, of which 21 were rejected (Additional file 1: Appendix 2). Hence, only one study from the initial systematic search met the inclusion criteria [34]. Subsequent citation searching and reference scanning identified a further six studies (N = 6) which met the inclusion criteria. A total of seven studies (N = 7) were included in our review. Included studies are referenced in Table 1, and a full bibliography is available in Additional file 1: Appendix 3.

**Table 1:** Quality assessment (mNOS) of identified studies

No	Author and year	Quality assessment (modified NOS)	Grade
1	Arora et al. [35]	4	Satisfactory
2	Berridge et al. [34]	2	Unsatisfactory
3	Bu et al. [36]	4	Satisfactory
4	Eisen et al. [37]	2	Unsatisfactory
5	Pal et al. [38]	3	Satisfactory
6	Webb et al. [39]	2	Unsatisfactory
7	Wells et al. [40]	3	Satisfactory

We acknowledge that more studies were identified through reference scanning and citation search than through the electronic database search. This could be due to the fact that the electronic database search terms included location phrases such as “United Kingdom” or “England”, whereas many of the included studies do not mention location in the title or abstract despite being indexed in the included databases.

### Quality assessment

Table 1 presents the mNOS scores for the included studies. Quality assessment identified the included studies as unsatisfactory with a score of two [34, 37, 39]; or satisfactory with scores of three [38, 40] or four [35, 36]. No studies scored good or very good.

Studies frequently lost points for a variety of reasons. The majority of studies failed to truly be representative of the average target population, because they mostly selected participants by convenience sampling. As the majority of the included studies were feasibility or pilot studies, the use of convenience sampling is understandable. Furthermore, most of the studies failed to justify their sample size. Although one study provided a summary of feedback received by non-participants [39], none provided the summary characteristics of non-respondents. This could result in participants who are not benefiting from the intervention dropping out, falsely improving end-outcomes. Also, none of the studies identified or adjusted for confounders.

### Sample and design

Table 2 presents details about the sample and design of the included studies. Sample size was reported by all studies (N = 7) and ranged widely from six to 150. Most studies (N = 5) did not explicitly justify their sample size. Two studies (N = 2) provided a flowchart justifying the number of included participants [39], with one stating that participant numbers had to be capped due to limited capacity to provide the intervention [36]. Only two studies (N = 2) reported the gender of participants [36], with more male than female participants reported in one [34].

**Table 2:** Sample and design of identified studies

No	Author and year	Sample size	Gender	Grade	Department	Methodology	Type of study	Recruitment strategy
1	Arora et al. [35]	18	Unspecified	FY1/FY2 18	Surgery 18	Quantitative (randomised-controlled design)	Pilot	Random
2	Berridge et al. [34]	50	Males 33, females 17	FY1 50	Medicine 30, surgery 18, unspecified 2	Mixed-methods	Longitudinal	Convenient
3	Bu et al. [36]	20	Males 10, females 10	FY1 10, FY2 10	Unspecified 20	Mixed-methods	Pilot	Convenient
4	Eisen et al. [37]	44	Unspecified	ST1 44	Paediatrics 44	Quantitative (randomised-controlled design)	Pilot	Random
5	Pal et al. [38]	6	Unspecified	Unspecified 6	Paediatrics 6	Quantitative	Pilot	Unspecified
6	Webb et al. [39]	42	Unspecified	CT1 21, CT2 21	Medicine 42	Quantitative	Pilot	Convenient
7	Wells et al. [40]	150	Unspecified	FY1 150	Unspecified 150	Quantitative	Longitudinal	Convenient

Doctors enter the Foundation Programme immediately after qualifying. After completing two years of this programme, they may apply for further core/speciality training (both of which follow the same scales i.e. ST1 is equivalent to CT1)

FY1/2, Foundation Programme Year 1/2; ST1, Speciality Trainee Year 1; CMT1/2, Core Trainee Year 1/2

Grades of participants ranged from FY1 to CT2 three studies (N = 3) did not explicitly state the grade of their participants in their paper [35, 37, 38], but authors from two studies confirmed that they were newly-qualified doctors within the first five years of graduation [35, 37]. Most of these studies (N = 4) were performed exclusively on Foundation doctors; two of these studies were conducted on participants during FY1 induction, meaning these participants had not started working as FY doctors yet [34, 40]. Most studies (N = 5) reported the specialty where the doctors were working: these include paediatrics (N = 2), medicine (N = 1) [37–39], mixture of medicine and surgery (N = 1) [34].

Although all included studies presented quantitative data, two (N = 2) used mixed methods, hence reporting both quantitative and qualitative data [34, 36]. Two quantitative studies (N = 2) also gathered qualitative data using open-ended questions within the questionnaire, but did not report the analysis of this qualitative data [37, 39].

Five studies (N = 5) were pilot studies, while two (N = 2) were longitudinal studies. Four of the five pilot studies were small-scale studies, with the potential effectiveness, feasibility and acceptability reported. Additionally, we did not find any evidence of the five pilot studies being implemented as full studies at the time this systematic review was conducted.

All studies reported their recruitment strategy. However, we found the recruitment strategy of one study (N = 1) to be unclear because the recruitment strategy was not stated [38]. Four studies (N = 4) used convenient sampling. The remaining two studies (N = 2) stated they used random sampling, but did not randomly select potential participants [35, 37]. Arora et al. was the only study to use a prospective randomised-controlled design where participants were randomised to intervention or control [35].

As studies were volunteer-based, it is likely that the recruited participants were highly self-aware, willing to engage with these wellbeing interventions, and already had good motivation. Hence, the authors are unable to account for selection and non-response bias. The widespread convenience sampling is also unlikely to be representative of the larger junior doctor cohort.

### Interventions performed

Table 3 outlines the wellbeing interventions performed by the included studies. The interventions described varied significantly from one another and were characterised as either mindfulness courses (N = 3), clinical preparation courses (N = 2) or mentorship programmes (N = 2). The clinical preparation courses were performed on newly-qualified doctors as an induction programme before clinical work commenced; these two papers also collected data in three stages [34, 40]. Both studies using mentorship programmes collected data over an entire year, representing the longest follow-up times amongst the included studies in this systematic review [37, 39]. Given that the follow-up time in these interventions was relatively short, the duration for which the outcome lasts may be overestimated due to impact bias. Finally, the two mindfulness courses involved multiple sessions of mindfulness training [36, 38]. One study specified this intervention to be conducted with a partner organisation Breathworks [36], while the other did not specify any details about their mindfulness intervention [38].

**Table 3:** Wellbeing interventions performed by included studies

No	Author and year	Duration of data collection	Stages for data collection	Screening tool	Intervention	Duration of intervention	Wellbeing outcomes measured	Non-wellbeing outcomes measured
1	Arora et al. [35]	5 days	2 stages: pre, post	ISAT (HR, salivary cortisol, STAI (6 item Likert scale))	Mental practice	2.5 h (0.5 h × 5 sessions)	Stress, anxiety	Mental imagery
2	Berridge et al. [34]	1 month	3 stages: pre, intermediate, post	Unspecified [17 item questionnaire used]	Preparation for Practice Course	2 weeks	Anxiety	Confidence, preparedness
3	Bu et al. [36]	3 months	2 stages: pre, post	Unspecified [1 item questionnaire used]	Mindfulness course delivered by Breathworks	12 h (2 h × 6 weeks)	Stress, overall wellbeing	*None specified*
4	Eisen et al. [37]	1 year	2 stages: pre, post	Unspecified (questionnaire used, details not given)	Mentorship programme with senior trainees	1 year (unstructured mentoring)	Stress management, work-life balance	Demand for course, perceived value of programme, self-confidence, transferable skill acquisition
5	Pal et al. [38]	Unspecified	2 stages: pre, post	GHQ12	Mindfulness course; email support between sessions	5 h (2.5 h × 2 sessions)	Anxiety	*None specified*
6	Webb et al. [39]	1 year	2 stages: pre, post	Unspecified (questionnaire used, unknown number of items)	Mentorship programme with second-year trainees	1–2.5 h (0.5 h × 2–5 sessions)	Confidence, work-life balance	Transferable skill acquisition, time management
7	Wells et al. [40]	8 months	3 stages: pre, 2 months post, 6 months post	Unspecified (7 item questionnaire used, details given)	Undergoing assistantship prior to starting FY2	1 month	Anxiety	Confidence, preparedness, perceived value of programme

ISAT, Imperial Stress Assessment Tool; STAI, State Trait Anxiety Inventory; h, hour(s); GHQ12, General Health Questionnaire 12; FY2, Foundation Programme Year 2

The total duration of interventions in this systematic review lasted between one hour and twelve hours. Three studies (N = 3) did not clearly state the total number of hours over which the interventions were run [34, 37, 40]. Instead, only the total duration for data collection was indicated.

Duration of data collection typically corresponded to duration of the intervention. Data collection duration lasted between five days and one year; one study (N = 1) did not specify duration of data collection [38]. Most studies (N = 5) collected data at two stages: before and after the intervention, while two studies (N = 2) collected data at three intervals: before the intervention, after the intervention, and in the interim [34] or six months after the intervention commenced [40]. Just one study (N = 1) reported details of non-respondents and participant flow through the study [39].

Only two studies (N = 2) used validated tools to screen for or measure wellbeing outcomes: the General Health Questionnaire 12 (GHQ12) [38] and the Imperial Stress Assessment Tool (which includes objective measures of stress) were used respectively [35]. The remaining studies (N = 5) produced their own questionnaires; it is unclear whether or not the questionnaires were validated. All questionnaires involved rating on a Likert-scale, although questionnaire details were not provided in one study [37].

### Results from interventions

Table 4 summaries the results from the included studies, and states the statistical analytical tools used by each. Majority of interventions measured anxiety (N = 3) or stress (N = 3) as their intended outcome, while one study (N = 1) did not specify their intended outcome [38]. Only one study (N = 1) explicitly measured both anxiety and stress as primary intended outcomes [35]. The remaining studies (N = 6) reported stress or anxiety as secondary outcomes either alongside the primary aims of the study, or incidentally while collecting data.

**Table 4:** Results and statistical analysis of included studies

No	Author and year	Results	Statistical analysis
1	Arora et al. [35]	Decreased subjective anxiety (mean STAI 8.40 in intervention vs 11.31 in control). Decreased objective stress (mean HR 77 vs 88 bpm, max HR 102 vs 119 bpm and cortisol 2.25 vs 3.85 nmol/L)	Mann–Whitney U test performed. Decreased mean anxiety and stress statistically significant (*p* < 0.05). Confidence intervals not reported
2	Berridge et al. [34]	Decreased anxiety, including physical symptoms of anxiety (mean from 3.04 to 4.00 out of 5.00 on an inverted scale). Improved confidence and preparedness	Mann–Whitney U test performed. Improved mean confidence was statistically significant. Decreased anxiety was not statistically significant. Confidence intervals not reported
3	Bu et al. [36]	Decreased stress (median from 6.5 to 5.0 out of 10.0). Doctors reported being more mindful and having improved overall wellbeing	Mann–Whitney U test performed. Decreased median stress statistically significant (*p* = 0.04). Confidence intervals not reported
4	Eisen et al. [37]	Improved stress management and work-life balance (78% of participants)	Statistical significance of effect measure (binary outcome) and confidence intervals not reported
5	Pal et al. [38]	No difference in GHQ12 over the course. All participants enjoyed the course	Unspecified statistical test. Results not statistically significant (*p* = 0.43) with wide standard deviation of results
6	Webb et al. [39]	Improvement in stress management and work-life balance (not reported)	Statistical significance of effect measure (binary outcome) and confidence intervals not reported
7	Wells et al. [40]	Improved anxiety relief during first placement (mean from 3.9 to 4.1 out of 5.0). However, this improvement had disappeared by the last stage of data collection (mean 3.3)	ANOVA performed. Mean anxiety relief statistically significant at first placement (*p* < 0.03), but not second placement. Confidence intervals not reported

STAI, State Trait Anxiety Inventory; HR, heart rate; GHQ12, General Health Questionnaire 12; ANOVA, repeated measures analysis of variance

Four studies (N = 4) provided P-values and reported effect sizes as changes to mean or median assessment scores, although none reported confidence intervals. Three studies performed Mann–Whitney U test [34–36], while one performed repeated measures analysis of variance [40], and three were unspecified or unreported [37–39]. Pal et al. did not find statistically significant results for GHQ12, which screens for non-psychotic psychiatric conditions including anxiety; Berridge et al. found that anxiety relief was not statistically significant; and Wells et al. found that anxiety relief was no longer statistically significant by the third stage of data collection. The identified quantitative data were typically composed of small sample sizes, varied outcomes and heterogeneous populations with often inadequately defined participant characteristics. Hence, meta-analysis and statistical analysis were not performed in this review.

All studies but one (N = 6) reported that wellbeing interventions improved stress management and anxiety. Mindfulness courses (N = 2), clinical preparation courses (N = 2) and mentorship programmes (N = 2) identified an improvement in stress or anxiety. Pal et al. was the only study (N = 1) which failed to identify an improvement in their measured outcome [38]. All studies except one explicitly reported the quantitative change produced by their intervention [39]. The studies which performed qualitative analysis (N = 2) reported findings which were convergent with quantitative results [34, 36].

Clinical preparation courses such as assistantship programmes following graduation, and mindfulness courses such as mental practice were reported to improve anxiety in participants [34, 35, 40]. All studies noted improvements to subjective self-reported anxiety scores. However, Wells et al. noted that the anxiety relief provided by their programme was only valid at the first rotation and was subsequently not valid when data was collected in the next rotation [40]; the other two studies did not measure outcomes beyond one month. Additionally, Pal et al. failed to state the wellbeing outcome being measured, but noted no statistically significant difference in GHQ12 score (used to screen for non-psychotic morbidity) following a mindfulness intervention.

Studies indicated that mindfulness courses, mental practice and mentorship quantitatively improve stress levels [35–37]. One mentorship programme failed to provide any quantitative data but wrote that their intervention improved stress management and work-life balance [39]. The majority of included papers (N = 4) were pilot studies, of which three (N = 3) demonstrated a positive result.

## Discussion

This systematic review reports quantitative evidence on interventions aimed to improve junior doctor wellbeing since the introduction of the Foundation Programme in the UK. In this, we identified a number of wellbeing interventions for newly-qualified doctors such as mentorship, mindfulness and clinical preparation interventions. Remarkably, improvements in wellbeing were identified, demonstrating the effectiveness of these interventions by a number of studies. This is consistent with findings from previous systematic reviews conducted on physicians, where mindfulness training was identified as being beneficial to wellbeing [21, 23]. However, no study in our systematic review used interventions such as yoga or cognitive therapy to improve junior doctor wellbeing, as documented in earlier systematic reviews including studies performed in other countries [21, 24].

It is widely recognised that Randomised Control Trials (RCTs) are the gold standard to demonstrate effectiveness of an intervention [41]. However, we acknowledge that RCT may not always be plausible. In the case of newly-qualified doctors, randomisation will affect the consistency and accountability of their training [41]. Nevertheless, methodically robust studies will still provide more generalisable results which can demonstrate the effectiveness of interventions.

In this review, many included studies were small-scale pilot studies using convenience sampling; hence, the results cannot be deemed representative or generalisable. Additionally, studies provided limited details on effect sizes or statistical significance. Performing objective assessment using mNOS allowed us to better quantify the quality of these included studies, which makes this conclusion on generalisability more robust. These findings are consistent with previous systematic reviews, some of which explicitly identify the necessity for improved methodology in research on interventions which improve physician wellbeing [22, 23]. However, a systematic review performed in the United States did identify a number of RCTs comparing the effects of cognitive therapies to no intervention on the wellbeing of healthcare workers, and found the cognitive therapies to be successful at improving wellbeing compared to no intervention [21].

It is unclear whether all the studies which were included in this review reported all possible outcomes, or whether only positive outcomes were presented. Hence, the authors are unable to account for publishing and reporting bias. This contradicts the findings identified in an earlier systematic review by Panagioti et al., where a meta-analysis on wellbeing intervention studies performed on healthcare workers did not observe any indications of publication bias amongst the included studies [19].

Furthermore, previous research on junior doctor wellbeing lacks consistency in measuring wellbeing, with no clear consensus or widespread use of validated tools. The wide variety of wellbeing interventions raises the difficulty of establishing a common denominator relevant to all studies which can be quantitatively measured. Previous systematic reviews which include studies performed outside the UK have also identified the difficulty in objective measurement of wellbeing-related outcomes [22].

A limitation of this review process is that the majority of included studies were identified at reference or citation search, suggesting that the systematic search may not have been sufficiently inclusive. This is likely due to most of the papers not explicitly stating the country in which the study was performed. Therefore, there is the risk that this review has missed studies not indexed by citation search databases. This review did not include grey literature, which may be the dissemination method for some locally-run wellbeing interventions. Additionally, bias was not formally assessed, as we did not evaluate missingness of results in included studies.

A major gap in research about wellbeing interventions has been identified, as none of the studies indicated that wellbeing interventions took into cognisance job-related factors. A 2019 qualitative study from the Republic of Ireland identified a number of job-related factors which may influence junior doctor wellbeing [17]. These factors include: inadequate staffing leading to difficulty accessing statutory leave; lack of time to spend on self-care; fear of professional consequence from seeking help; and poor support from managers. To improve junior doctor wellbeing, the authors recommend: spare staffing capacity including implementation of “floating staff members”; development of clinical management skills; debriefing; cultivating interests outside work; and a change in culture from competition to compassion.

This view is also shared by previous systematic reviews performed outside the UK, where other interventions such as organisation-directed changes (including rescheduling rotas and reducing workload) and psychosocial skills training have been helpful in improving wellbeing of doctors or fostering resilience in physicians [19, 23]. Additionally, organisational changes in other countries have been identified as more effective in improving physician wellbeing when compared to the interventions documented in UK-based studies [19, 24]. Some authors are now recommending that in addition to wellbeing interventions, other organisational protective strategies such as increasing departmental staffing, good leadership and diversification of activities (including teaching and research) should be considered [42].

Moreover, the recent COVID-19 pandemic has also demonstrated that wellbeing interventions need to be holistic because factors impacting on the wellbeing of newly-qualified doctors are not only inherent in the FY and CT training programmes, but may equally be organisationally-related. For instance, many doctors were redeployed or placed on emergency rotas during the pandemic. In such scenarios, interventions such as mindfulness, clinical preparation or mentorship programmes will not suffice in improving junior doctor wellbeing. It is believed that addressing these organisational related factors may help address the root causes which influence junior doctor wellbeing.

## Conclusion

Junior doctors’ wellbeing should be prioritised in the UK as they are at a high risk of burnout. Hence, it is imperative to identify interventions which are effective and equally cost-effective. However, this review confirms the current dearth of evidence on the effectiveness of interventions, and the limited information available to organisations attempting to select an appropriate mechanism to support their newly-qualified doctors.

A recommendation for future research is for subsequent studies to design and implement large scale interventions using appropriate research design and a representative sample that will make the results generalisable. In addition, we will suggest the use of a holistic model when designing interventions for newly-qualified doctors, to address the various institutional and personal factors involved in junior doctor burnout. This will ensure that intervention programmes take into cognisance all the factors which impact on the wellbeing of newly-qualified doctors.

A limitation of this systematic review is that most of the included studies were small scale pilot studies. Regardless, this review is the first to systematically identify and appraise studies performed on junior doctor wellbeing in the UK since the introduction of the Foundation Programme and identifies the necessity for gathering high-quality evidence on the myriad of interventions currently being implemented across hospitals in the UK.

## Supplementary Information

**Additional file 1**. Appendices.

**Additional file 2**. Data extraction and summary tables.

## Data Availability

The datasets used and/or analysed during the current study are available from the corresponding author on reasonable request.

## Abbreviations

ANOVA: Analysis of variance
bpm: Beats per minute
CT: Core trainee (doctor)
FY: Foundation Year (doctor)
GHQ12: General Health Questionnaire 12
HR: Heart rate
mNOS: Modified Newcastle-Ottawa Scale
NHS: National Health Service
PRISMA: Preferred reporting items for systematic reviews and meta-analyses
RCT: Randomised controlled trial
STAI: State Trait Anxiety Inventory
UK: United Kingdom
UKFP: United Kingdom Foundation Programme
