# Supplementary material for: A systematic review of interventions aiming to improve newly-qualified doctors’ wellbeing in the United Kingdom
Source: BMC Psychol. 2022 Jun 26;10:161. doi: 10.1186/s40359-022-00868-8 (PMC9235154; doi:10.1186/s40359-022-00868-8)
Supplement: Supplementary file 1 — Additional file 1. Appendices. [file 40359_2022_868_MOESM1_ESM.docx]

Appendices

# Appendix 1 - Search strategies

| **Database** | **Search strategy** |
| --- | --- |
| EMBASE | ((((("employee assist* programme*" OR "coping mechanism*" OR intervention OR support* OR "Occupational Health" OR welfare OR wellbeing OR "Psychological adj3 Wellbeing*" OR “mindful*” OR “Balint*” OR “Schwartz round*” OR psychotherap*).ti,ab OR "BALINT GROUP"/ OR "SUPPORT GROUP"/ OR PSYCHOTHERAPY/ OR "QUALITY OF WORKING LIFE"/ OR "PSYCHOLOGICAL WELL-BEING"/ OR “MINDFULNESS”/ OR "PSYCHOLOGICAL WELLBEING ASSESSMENT"/) AND (("Mental disorder*" OR suicid* OR resilien* OR "Self-Harm*" OR "Mental Illness*" OR "low morale" OR stress* OR "Mental* health*" OR "mental condition*" OR Burnout OR "work* stress" OR "occupation* stress").ti,ab OR "MENTAL DISEASE"/ OR SUICIDE/ OR "PSYCHOLOGICAL RESILIENCE"/ OR AUTOMUTILATION/ OR "PROFESSIONAL BURNOUT"/ OR "EMOTIONAL STRESS"/ OR "DEPRESSION ANXIETY STRESS SCALE"/ OR "PSYCHOLOGICAL WELL-BEING"/ OR "JOB STRESS"/)) AND ((Doctor* OR "Junior Doctor*" OR "train* doctor" OR "core train*" OR "foundation year*" OR "foundation program*" OR “medical gradua*”).ti,ab OR PHYSICIAN/)) AND ((United Kingdom OR Scotland OR England OR Great Britain).ti,ab OR "UNITED KINGDOM"/ OR (Wales NOT south).ti,ab OR ("Northern Ireland" OR (Ireland NOT republic)).ti,ab)) [DT 2004-2019] [Languages English] |
| PsycINFO | ((((("employee assist* programme*" OR "coping mechanism*" OR “intervention” OR support* OR "Occupational Health" OR welfare OR wellbeing OR "Psychological adj3 Wellbeing*" OR “mindful*” OR “Balint*” OR “Schwartz round*” OR psychotherap*).ti,ab OR PSYCHOTHERAPY/ OR "COPING BEHAVIOR"/ OR “MINDFULNESS”/ OR "SOCIAL SUPPORT"/ OR "OCCUPATIONAL HEALTH"/) AND (("Mental disorder*" OR suicid* OR resilien* OR "Self-Harm*" OR "Mental Illness*" OR "low morale" OR stress* OR "Mental* health*" OR "mental condition*" OR Burnout OR "work* stress" OR "occupation* stress").ti,ab OR "MENTAL DISORDERS"/ OR SUICIDE/ OR "RESILIENCE (PSYCHOLOGICAL)"/ OR "MENTAL HEALTH AND ILLNESS ASSESSMENT"/ OR "OCCUPATIONAL STRESS"/)) AND ((Doctor* OR "Junior Doctor*" OR "train* doctor" OR "core train*" OR "foundation year*" OR "foundation programme" OR “medical gradua*”).ti,ab OR PHYSICIANS/)) AND ((United Kingdom OR Scotland OR England OR Great Britain).ti,ab OR (Wales NOT south).ti,ab OR ("Northern Ireland" OR (Ireland NOT republic)).ti,ab)) [DT 2004-2019] [Languages English] |
| PubMed | ((("employee assist* programme*" OR "coping mechanism*" OR intervention OR support* OR "Occupational Health" OR welfare OR wellbeing OR "Psychological adj3 Wellbeing*" OR “mindful*” OR “Balint*” OR “Schwartz round*” OR psychotherap*).ti,ab AND ("Mental disorder*" OR suicid* OR resilien* OR "Self-Harm*" OR "Mental Illness*" OR "low morale" OR stress* OR "Mental* health*" OR "mental condition*" OR Burnout OR "work* stress" OR "occupation* stress").ti,ab) AND (Doctor* OR "Junior Doctor*" OR "train* doctor" OR "core train*" OR "foundation year*" OR "foundation programme" OR “medical gradua*”).ti,ab) AND ((United Kingdom OR Scotland OR England OR Great Britain).ti,ab OR (Wales NOT south).ti,ab OR ("Northern Ireland" OR (Ireland NOT republic)).ti,ab) |
| CINAHL | ((((("employee assist* programme*" OR "coping mechanism*" OR intervention OR support* OR "Occupational Health" OR welfare OR wellbeing OR "Psychological adj3 Wellbeing*" OR “mindful*” OR “Balint*” OR “Schwartz round*” OR psychotherap*).ti,ab OR "OCCUPATIONAL HEALTH SERVICES"/ OR PSYCHOTHERAPY/ OR "SELF-RATING DEPRESSION SCALE"/ OR “MINDFULNESS”/ OR "SELF-RATING ANXIETY SCALE"/ OR "PERLOW SELF-ESTEEM SCALE"/ OR "HAMILTON RATING SCALE FOR DEPRESSION"/ OR COPING/ OR "OCCUPATIONAL HEALTH"/ OR "PSYCHOLOGICAL WELL-BEING"/) AND (("Mental disorder*" OR suicid* OR resilien* OR "Self-Harm*" OR "Mental Illness*" OR "low morale" OR stress* OR "Mental* health*" OR "mental condition*" OR Burnout OR "work* stress" OR "occupation* stress").ti,ab OR "MENTAL DISORDERS"/ OR SUICIDE/ OR "INJURIES, SELF-INFLICTED"/ OR STRESS/ OR "STRESS, OCCUPATIONAL"/ OR "BURNOUT, PROFESSIONAL"/)) AND ((Doctor* OR "Junior Doctor*" OR "train* doctor" OR "core train*" OR "foundation year*" OR "foundation programme" OR “medical gradua*”).ti,ab OR PHYSICIANS/)) AND ((United Kingdom OR Scotland OR England OR Great Britain).ti,ab OR "UNITED KINGDOM"/ OR (Wales NOT south).ti,ab OR ("Northern Ireland" OR (Ireland NOT republic)).ti,ab)) [DT 2004-2019] [Languages eng] |
| Medline | (((("employee assist* programme*" OR "coping mechanism*" OR intervention OR support* OR "Occupational Health" OR welfare OR wellbeing OR "Psychological adj3 Wellbeing*" OR “mindful*” OR “Balint*” OR “Schwartz round*” OR psychotherap*).ti,ab OR "PSYCHOANALYTIC THERAPY"/ OR PSYCHOTHERAPY/ OR "ADAPTATION, PSYCHOLOGICAL"/ OR “MINDFULNESS”/ OR "OCCUPATIONAL HEALTH"/) AND (("Mental disorder*" OR suicid* OR resilien* OR "Self-Harm*" OR "Mental Illness*" OR "low morale" OR stress* OR "Mental* health*" OR "mental condition*" OR Burnout OR "work* stress" OR "occupation* stress").ti,ab OR "MENTAL DISORDERS"/ OR "OCCUPATIONAL STRESS"/ OR "STRESS, PSYCHOLOGICAL"/ OR "BURNOUT, PSYCHOLOGICAL"/)) AND ((Doctor* OR "Junior Doctor*" OR "train* doctor" OR "core train*" OR "foundation year*" OR "foundation programme" OR “medical gradua*”).ti,ab OR PHYSICIANS/)) AND ((United Kingdom OR Scotland OR England OR Great Britain).ti,ab OR "UNITED KINGDOM"/ OR (Wales NOT south).ti,ab OR ("Northern Ireland" OR (Ireland NOT republic)).ti,ab) |

##

# Appendix 2 - Studies rejected at full-article screening

| **No.** | **Study details** | **Reason** | **Comments** |
| --- | --- | --- | --- |
| 1 | Taylor C, Xyrichis A, Leamy MC, Reynolds E, Maben J. Can Schwartz Center Rounds support healthcare staff with emotional challenges at work, and how do they compare with other interventions aimed at providing similar support? A systematic review and scoping reviews. BMJ open. 2018 Oct 1;8(10):e024254. | Not UK | None of the studies are relevant to our inclusion criteria i.e. not the UK. |
| 2 | Dowling S, Last J, Finnegan H, Daly P, Bourke J, Hanrahan C, Harrold P, McCombe G, Cullen W. Impact of participation in continuing medical education small group learning (CME-SGL) on the stress, morale, and professional isolation of rurally-based GPs: a qualitative study in Ireland. BJGP open. 2019 Dec 1;3(4). | Wrong population | Small group learning intervention run on rural GPs to improve wellbeing (not junior doctors). |
| 3 | McKimm J, Vogan CL, Roberts C, Nash E, Hothersall E, Jones PK. The Swansea 6D model: a diagnostic and conversational framework for supervisors, mentors and doctors in training. Postgraduate Medical Journal. 2019 Sep 1;95(1127):482-6. | No intervention | Produced a model to improve junior doctor wellbeing — did not put it into action? |
| 4 | Leamy M, Reynolds E, Robert G, Taylor C, Maben J. The origins and implementation of an intervention to support healthcare staff to deliver compassionate care: exploring fidelity and adaptation in the transfer of Schwartz Center Rounds® from the United States to the United Kingdom. BMC health services research. 2019 Dec;19(1):1-1. | No intervention | Adapting US-based intervention to the UK. Did not actively measure any outcomes related to the intervention. |
| 5 | Rizan C, Montgomery J, Ramage C, Welch J, Dewhurst G. Why are UK junior doctors taking time out of training and what are their experiences? A qualitative study. Journal of the Royal Society of Medicine. 2019 May;112(5):192-9. | Intervention not validated | Qualitative evidence for FY3 year doctors. |
| 6 | Radia T, Depani S, Williams N, Cooper M, Kingdon C. G456 (P) Mindful and supported return to clinical practice for paediatric trainees. | Wrong time frame | Does not specify the grade of trainees. |
| 7 | Lynch J, Prihodova L, Dunne PJ, O’Leary C, Breen R, Carroll Á, Walsh C, McMahon G, White B. Mantra meditation programme for emergency department staff: a qualitative study. BMJ open. 2018 Sep 1;8(9):e020685. | Qualitative | Qualitative arm of an RCT. DId not include junior doctors, looked at other members of the MDT at A&E in Ireland. |
| 8 | Beresford B, Gibson F, Bayliss J, Mukherjee S. Preventing work‐related stress among staff working in children's cancer Principal Treatment Centres in the UK: a brief survey of staff support systems and practices. European Journal of Cancer Care. 2018 Mar;27(2):e12535. | Wrong outcome measured | Surveyed into available services. Not limited to junior doctors. No outcome measured. |
| 9 | Wainwright E, Fox F, Breffni T, Taylor G, O’Connor M. Coming back from the edge: a qualitative study of a professional support unit for junior doctors. BMC medical education. 2017 Dec;17(1):1-1. | Qualitative | Impact of physician support units on UK junior doctors — no outcome measured, qualitative study. |
| 10 | Hameed Y, De Waal H, Bosier E, Miller J, Still J, Collins D, Bennet T, Haroulis C, Hamelijnck J, Gill N. Using mentoring to improve the foundation placement in psychiatry: review of literature and a practical example. British Journal of Medical Practitioners. 2017;9(4):a932. | Wrong outcome measured | No wellbeing outcome measured. |
| 11 | Gerada C. Healing doctors through groups: creating time to reflect together. British Journal of General Practice. 2016 Oct 1;66(651):e776-8. | No intervention | Explains services but does not measure outcomes following a particular intervention. Not limited to junior doctors. |
| 12 | Austen L. Increasing emotional support for healthcare workers can rebalance clinical detachment and empathy. British Journal of General Practice. 2016 Jul 1;66(648):376-7. | No intervention | Letter from a GP about thoughts of empathy and emotional support for healthcare workers. No intervention, no outcome measured. Not limited to junior doctors. |
| 13 | Braquehais MD, Tresidder A, DuPont RL. Service provision to physicians with mental health and addiction problems. Current Opinion in Psychiatry. 2015 Jul 1;28(4):324-9. | Wrong outcome measured | No outcome measured. Not limited to UK or junior doctors. |
| 14 | Hassan TB. Sustainable working practices and minimizing burnout in emergency medicine. British Journal of Hospital Medicine. 2014 Nov 2;75(11):617-9. | No intervention | No intervention performed. |
| 15 | Moonesinghe SR, Lowery J, Shahi N, Millen A, Beard JD. Impact of reduction in working hours for doctors in training on postgraduate medical education and patients’ outcomes: systematic review. Bmj. 2011 Mar 22;342. | Wrong outcome measured | No wellbeing outcome measured; measure patient outcomes instead. Not limited to the UK. |
| 16 | Brooks SK, Chalder T, Gerada C. Doctors vulnerable to psychological distress and addictions: treatment from the Practitioner Health Programme. Journal of Mental Health. 2011 Apr 1;20(2):157-64. | Wrong population | Does not make any reference to junior doctors. |
| 17 | Field R, Haslam D. Do you have your own doctor, doctor? Tackling barriers to health care. British Journal of General Practice. 2008 Jul 1;58(552):462-4. | No intervention | No intervention, no outcome measured. |
| 18 | Feld J, Heyse-Moore L. An evaluation of a support group for junior doctors working in palliative medicine. American Journal of Hospice and Palliative Medicine®. 2006 Aug;23(4):287-96. | Wrong time frame | Study was performed before 2002. |
| 19 | Peterson A, Monaghan H. Near-peer mentorship: a pilot programme to improve support for new doctors. BMJ Leader. 2019 Feb 16:leader-2018. | Wrong outcome measured | Does not measure quantitative outcome; does not measure wellbeing/stress outcome; describes set up process of the pilot study |
| 20 | Hall LH, Johnson J, Watt I, Tsipa A, O’Connor DB. Healthcare staff wellbeing, burnout, and patient safety: a systematic review. PloS one. 2016 Jul 8;11(7):e0159015. | Wrong population | Systematic review which only includes one UK study which is on pharmacists, not doctors. |
| 21 | Imo UO. Burnout and psychiatric morbidity among doctors in the UK: a systematic literature review of prevalence and associated factors. BJPsych bulletin. 2017 Aug;41(4):197-204. | No intervention | No intervention performed; systematic review of causes of psychiatric morbidity in doctors. |

#

# Appendix 3 - Studies included in the systematic review

| **No.** | **Study details** |
| --- | --- |
| 1 | Arora S, Aggarwal R, Moran A, Sirimanna P, Crochet P, Darzi A, Kneebone R, Sevdalis N. Mental practice: effective stress management training for novice surgeons*. J.Am.Coll.Surg.* 2011; **212**: 225-33. |
| 2 | Berridge E, Freeth D, Sharpe J, Roberts CM. Bridging the gap: supporting the transition from medical student to practising doctor–a two-week preparation programme after graduation*. Med.Teach.* 2007; **29**: 119-27. |
| 3 | Bu CNN, Cotzias E, Panagioti M. Mindfulness intervention for foundation year doctors: a feasibility study*. Pilot and Feasibility Studies* 2019; **5**: 61. |
| 4 | Eisen S, Sukhani S, Brightwell A, Stoneham S, Long A. Peer mentoring: evaluation of a novel programme in paediatrics*. Arch.Dis.Child.* 2014; **99**: 142-6. |
| 5 | Pal S, Kelsall AW. Physician, Heal Thyself: Introduction and Evaluation of Pilot Mindfulness Short Course for Junior Doctor*. Archives of Disease in Childhood* 2017. |
| 6 | Webb J, Brightwell A, Sarkar P, Rabbie R, Chakravorty I. Peer mentoring for core medical trainees: uptake and impact*. Postgrad.Med.J.* 2015; **91**: 188-92. |
| 7 | Wells SE, Bullock A, Monrouxe LV. Newly qualified doctors’ perceived effects of assistantship alignment with first post: a longitudinal questionnaire study*. BMJ Open* 2019; **9**: e023992. |

Appendix 4 - Data extraction

Please see attached spreadsheet for full data extraction.

| **Ref** | **Author and year** | **Sample size** | **Type of study** | **Recruitment strategy** | **Methodology** | **Gender** | **Grade** | **Department** | **Screening tool** | **Intervention** | **Presence of pre-existing condition** | **Duration of data collection** | **Duration of intervention** | **Outcomes measured** | **Stages for data collection** | **Results** | **Quality assessment (modified NOS)** | **Grade** |
| --- | --- | --- | --- | --- | --- | --- | --- | --- | --- | --- | --- | --- | --- | --- | --- | --- | --- | --- |
| 1 | Arora et al. 2011 | 18 | Pilot | Random | Quantitative | Unspecified | Unspecified; confirmed to be within first 2 years in correspondence | Surgery | Imperial Stress Assessment Tool (HR, salivary cortisol, State Trait Anxiety Inventory (6 item Likert scale)) | Mental practice | None | 5 days | 2.5 hours (0.5h x 5 sessions) | Imperial Stress Assessment Tool (HR, salivary cortisol, State Trait Anxiety Inventory (6 item Likert scale)) | 2: pre, post | Subjective stress lower for group completing mental practice; objective stress also reduced for mental practice (average HR 77 vs 88 bpm, max HR 102 vs 119 bpm and cortisol 2.25 vs 3.85 nmol/L) | 4 | Satisfactory |
| 2 | Berridge et al. 2007 | 50 | Longitudinal | Convenient, non-random | Mixed-methods | Males 33, females 17 | FY1 | Medicine 30, surgery 18, missing 2 | Unspecified (17 item questionnaire used) | Preparation for Practice Course | None | 1 month | 2 weeks | Anxiety, confidence in skills, preparedness | 3: pre, intermediate, post | Improved confidence in relation to clinical skills. Level of preparedness increased. No changes in anxiety level. | 2 | Unsatisfactory |
| 3 | Bu et al. 2019 | 20 | Pilot | Convenient, non-random | Mixed-methods | Males 10, females 10 | FY1 10, FY2 10 | All departments | Unspecified (1 item questionnaire used); self-reported | Mindfulness course delivered by Breathworks | None | 3 months | 12 hours (2h x 6 weeks) | Stress | 2: pre, post | Stress was significantly lower after the course compared to baseline (mean from 6.5 to 5.0). Doctors reported being more mindful and having improved overall wellbeing. 12/14 improved working life and relationship with patients | 4 | Satisfactory |
| 4 | Eisen et al. 2014 | 44 | Pilot | Convenient, random | Mixed-methods | Unspecified | First year paediatric trainees; confirmed to be ST1 in correspondence | Paediatrics | Unspecified (questionnaire used, details not given) | Mentorship programme with senior trainees | None | 1 year | 1 year (unstructured mentoring) | Stress management, work-life balance | 2: pre, post | Mentees reported improved stress management (78% of participants), and improved work-life balance. | 2 | Unsatisfactory |
| 5 | Pal et al. 2017 | 6 | Pilot | Unspecified | Quantitative | Unspecified | Unspecified | Paediatrics | GHQ12 | Mindfulness course | None | Unspecified | 5 hours (2.5h x 2 sessions) | None reported | 2: pre, post | No statistically significant difference in GHQ12 over the course. | 3 | Satisfactory |
| 6 | Webb et al. 2015 | 42 | Pilot | Convenient, non-random | Mixed-methods | Unspecified | First and second year core medical trainees | Medicine | Unspecified (questionnaire used, details given) | Mentorship programme with second-year trainees | None | 1 year | 1-2.5h (0.5h x 2-5 sessions) | Confidence, learning from session | 2: pre, post | Mentees reported improved work life balance. Both mentors and mentees reported improvement in managing stressful sitatuons. | 2 | Unsatisfactory |
| 7 | Wells et al. 2019 | 150 | Longitudinal | Convenient, non-random | Quantitative | Unspecified | FY1 | All departments | Unspecified (7 item questionnaire used, details given) | Undergoing assistantship prior to starting FY1 | None | 8 months | 1 month | Anxiety, confidence, preparedness | 3: pre, 2 months post (first placement), 6 months post (second placement) | Candidates who underwent assistantship reported significantly greater anxiety relief at T2 (first placement); but difference had disappeared by T3 (second placement) | 3 | Satisfactory |

#

# Appendix 5 - modified Newcastle-Ottawa Quality Assessment Scale (mNOS) rubric (33)

| **Domain** | **2 points** | **1 point** | **0 points** |
| --- | --- | --- | --- |
| ***Selection*** | | | |
| *Representativeness of the sample* |  | Random sampling truly represents the average in the target population; or  Non-random sampling somewhat representative of the target group | Convenience sample; or  No description of the derivation of the included subjects |
| *Sample size* |  | Justified and satisfactory | Not justified; or  Inadequate information provided |
| *Non-respondents* |  | Proportion of target sample recruited attains pre-specified target; or  Basic summary of non-respondent characteristics in sampling frame recorded | Unsatisfactory recruitment rate; or  No summary data on non-respondents; or  No information provided |
| ***Comparability*** | | | |
| *Confounding factors* | Data adjusted for relevant predictors/risk factors/confounders e.g. age or sex. |  | Data not adjusted for all relevant predictors/risk factors/confounders. |
| ***Outcome*** | | | |
| *Assessment* | Independent blind assessment using objective validated laboratory methods; or  Unblinded assessment using objective validated laboratory methods | Used non-standard/ non-validated laboratory methods with gold standard | No description; or  Non-standard laboratory methods used |
| *Statistical tests* |  | Appropriate statistical test used to analyse the data described; and  Measures of association presented including confidence intervals and probability level (p value) | Statistical test not appropriate, not described or incomplete |

# Appendix 6 - modified Newcastle-Ottawa Quality Assessment Scale (mNOS) scoring criteria (33)

| **Point range** | **Description** |
| --- | --- |
| 0 to 2 points | Unsatisfactory study quality |
| 3 to 4 points | Satisfactory study quality |
| 5 to 6 points | Good study quality |
| 7 to 8 points | Very good study quality |
